# Identification of New KCNT1‐Epilepsy Drugs by *In Silico*, Cell, and *Drosophila* Modeling

**DOI:** 10.1002/ana.78031

**Published:** 2025-09-13

**Authors:** Michael G. Ricos, Bethan A. Cole, Rashid Hussain, Grigori Y. Rychkov, Zeeshan Shaukat, Nadia Pilati, Stephen P. Muench, Katie J. Simmons, Leanne M. Dibbens, Jonathan D. Lippiat

**Affiliations:** ^1^ Epilepsy Research Group, Clinical and Health Sciences, Australian Centre for Precision Health University of South Australia Adelaide South Australia Australia; ^2^ School of Biomedical Sciences, Faculty of Biological Sciences University of Leeds Leeds UK; ^3^ School of Biomedicine University of Adelaide Adelaide South Australia Australia; ^4^ South Australian Health and Medical Research Institute Adelaide South Australia Australia; ^5^ BioTiChe Drug Discovery Srl, Istituto di Ricerca Pediatrica Citta' della Speranza Padova Italy; ^6^ Astbury Centre for Structural and Molecular Biology University of Leeds Leeds UK; ^7^ Present address: Department of Biochemical and Cellular Pharmacology, Genentech South San Francisco CA

## Abstract

**Objective:**

Hyperactive KCNT1 potassium channels, caused by gain‐of‐function mutations, are associated with a range of epilepsy disorders. Patients typically experience drug‐resistant seizures and, in cases with infantile onset, developmental regression can follow. KCNT1‐related disorders include epilepsy of infancy with migrating focal seizures and sleep‐related hypermotor epilepsy. There are currently no effective treatments for KCNT1 epilepsies, but suppressing overactive channels poses a potential strategy.

**Methods:**

Using the KCNT1 channel structure we in silico screened a library of known drugs for those predicted to block the channel pore to inhibit channel activity. Cellular KCNT1 channel inhibition was analyzed using electrophysiology and *Drosophila* bang‐sensitive assays were used to analyze seizure suppression. Brain penetration of one drug was analyzed using liquid chromatography–mass spectrometry in a mouse.

**Results:**

Eight known drugs were investigated in vitro for their effects on patient‐specific mutant KCNT1 channels, with 4 drugs showing significant reduction of K^+^ current amplitudes. The action of the 4 drugs was then analyzed in vivo and 2 were found to reduce the seizure phenotype in humanized *Drosophila* KCNT1 epilepsy models. One drug, antrafenine, was shown to cross the blood–brain barrier in mice.

**Interpretation:**

This study identified a known drug, antrafenine, that reduces KCNT1 channel activity, reduces seizure activity in *Drosophila*, and crosses the blood–brain barrier in the mouse, suggesting its potential applicability as a new treatment for KCNT1 epilepsy. The sequential in silico, in vitro, and in vivo mechanism‐based drug selection strategy used here may have broader application for other human disorders where a disease mechanism has been identified. ANN NEUROL 2025;98:1261–1274

Mutations in *KCNT1* are associated with a range of drug‐resistant epileptic and developmental neurological disorders. These include severe forms of epilepsy with onset in infancy, including epilepsy of infancy with migrating focal seizures (EIMFS) and later onset focal epilepsies, including sleep‐related hypermotor epilepsy.^1^, ^2^, ^3^, ^4^ The KCNT1 potassium channel subunit is expressed widely in the central nervous system and is activated primarily by intracellular sodium and weakly by depolarization.^5^, ^6^ *KCNT1* mutations are heterozygous missense changes in the KCNT1 potassium channel subunit and increase channel activity. Accumulating evidence indicates that reduced excitability of inhibitory neurons associated with *KCNT1* mutations may be involved in hyperexcitability and seizures.^7^, ^8^, ^9^, ^10^, ^11^, ^12^

Because *KCNT1*‐associated disorders involve increased KCNT1 channel activity in the central nervous system, its suppression is the basis of proposed therapeutics. Until recently, the only pharmacological agents known to inhibit KCNT1 channels were the nonselective cation channel inhibitors quinidine, bepridil, and clofilium,^13^, ^14^ each of which have potent effects on the cardiac action potential. Quinidine, a class 1a antiarrhythmic, has been assessed in *KCNT1*‐associated disorders, but therapeutically effective dosing is limited by its inhibition of cardiac ion channels and dangerous effects on the heartbeat.^15^ Because of this, attempts have been made to identify novel KCNT1 inhibitors that are more potent and selective over other ion channels.^16^, ^17^, ^18^ Although they are yet to reach clinical use, the efficacy of KCNT1 inhibitors in suppressing electrical activity in mouse models of epilepsy is particularly encouraging.^17^, ^18^, ^19^, ^20^ Clinically, there have been case reports of patients with KCNT1 epilepsy whose seizures have been reduced with the antitussive drugs tipepidine and dextromethorphan,^21^ the former of which has since been found to inhibit KCNT1 channels,^20^ and by fluoxetine, which, in addition to several other ion channels, has now been found to inhibit KCNT1.^22^, ^23^

We hypothesized that existing drugs could be identified that have previously unknown KCNT1 channel inhibition as an off‐target effect. We have previously utilized virtual high throughput screening to identify compounds predicted to occupy the pore in the structure of the chicken's KCNT1 channel, generated using cryogenic electron microscopy (cryo‐EM).^16^, ^24^ Because this approach successfully identified novel inhibitors of the human KCNT1 channel,^16^ we repeated this using a library of known drugs. Here, we describe in vitro KCNT1 inhibition as a novel property of 4 identified drugs and demonstrate the efficacy of 2 via in vitro and in vivo model systems. In both cellular and *Drosophila* models, we have used several common mutations found in patients with *KCNT1* epilepsy, involving different functional domains of the channel. Thus, these drugs may potentially be effective for a range of patients with different clinical phenotypes.

## Methods

### Virtual Screening and Drug Selection

The intracellular pore region of the chicken KCNT1 channel cryo‐EM structure (PDB: 5U70)^24^ assigned for docking studies was based upon the predicted binding of other inhibitors.^16^ A 25 Å clip of the structure around these residues was used as the receptor for docking using Glide (Schrödinger, Release 2020–2).^25^ The KCNT1 PDB file was prepared using the Protein Preparation Wizard in the Schrödinger Maestro Graphical User Interface (GUI). The academic version of the DrugBank library of known drug molecules (https://www.drugbank.com/academic_research) was downloaded and prepared using the OMEGA module^26^ of OpenEye software (OMEGA version 2.5.1.4; OpenEye Scientific Software) to produce energy‐minimized 3D structures before importing into Maestro GUI. The Glide standard precision screening mode was used to predict the binding pose of each ligand. Drugs were ranked based on predicted binding affinity, likely membrane permeability, and commercial availability. To predict blood–brain‐barrier permeability and central nervous system (CNS) activity, the drug structures were created in Maestro, treated with LigPrep using OPLS forcefield, and molecular descriptors were calculated using QikProp (all Schrödinger, release 2024–3).

### Molecular Biology

To replicate the heterozygous nature of *KCNT1*‐associated disorders and obtain channels comprising wild‐type (WT) and mutated subunits, a concatemeric approach was taken. To generate human *KCNT1* concatemers, plasmids termed donor and recipient were generated from the pcDNA6‐*KCNT1* construct used previously^16^ and empty pcDNA6 V5/His6 vector (Invitrogen) using standard polymerase chain reaction (PCR) and cloning techniques. The insert of the donor construct comprised the *KCNT1* coding sequence that lacked a start codon and preceded by bases encoding a GGGSGGGS linker. A second donor construct was generated by mutagenesis of this construct, replacing the linker sequence with “self‐cleaving” T2A motif from the *Thosea asigna* virus (GSGEGRGSLLTCGDVEENPG),^27^ which exhibits efficient cleavage in Chinese hamster ovary (CHO) cells.^28^ The recipient construct was generated by deleting the stop codon and introducing a unique XhoI site. Sequences containing disease mutation Y796H were subcloned into these constructs from a plasmid used previously.^16^ All sequences generated by PCR were confirmed by Sanger sequencing (Genewiz, Takeley, UK). Finally, the concatemeric construct was generated by subcloning the XhoI/AgeI fragment from the donor plasmid, containing the in‐frame linker and complete subunit sequence into the same sites in the recipient plasmid. Recombination‐deficient competent *Escherichia coli* (*E. coli*) cells and 32°C incubation temperature were used to reduce the frequency of deletions between identical sequences in the plasmid, which was confirmed by restriction analysis. For other experiments, constructs with WT, G288S, R398Q, or R928C mutant forms of *KCNT1* cDNA, tagged with YFP‐6His in the pCMV‐entry vectors, were described previously.^10^

### Cell Culture and Transfection

The CHO cells were cultured in Dulbecco's modified Eagle's Medium (ThermoFisher, UK) supplemented with 10% (v/v) fetal bovine serum, 50 U/ml penicillin and 0.05 mg/ml streptomycin, and incubated at 37°C in 5% CO_2_ atmosphere. For whole cell recording of WT/Y796H KCNT1, CHO cells were transiently co‐transfected with concatemeric WT/Y796H and EYFP, as described previously.^29^ For electrophysiological experiments, cells were plated onto borosilicate glass cover slips and used 2 to 4 days later. Human embryonic kidney 293T cells (HEK293T) were cultured in the same manner, but with medium supplemented with 2 mM L‐glutamine, and 1% (v/v) nonessential amino acids. For excised inside‐out recording of homomeric WT or mutant YFP‐tagged *KCNT1*, HEK293T cells plated on glass cover slips were transfected using Attractene Transfection Reagent (Qiagen, Germany) according to the manufacturer's instructions and used 2 to 3 days later.

### Electrophysiology

Whole cell recordings from transiently transfected CHO cells were conducted at room temperature and analyzed as described previously.^29^ The pipette (intracellular) solution contained, in mM, 100 K‐gluconate, 30 KCl, 10 Na‐gluconate, 29 glucose, 5 EGTA, and 10 HEPES, adjusted to pH 7.3 with KOH and the bath (extracellular) solution contained, in mM, 140 NaCl, 1 CaCl_2_, 5 KCl, 29 glucose, 10 HEPES, and 1 MgCl_2_, adjusted to pH 7.4 with NaOH. For current‐voltage and conductance‐voltage analysis of the concatemeric constructs, cells were held at −80 mV and 400 ms pulses were applied to voltages between −100 and 80 mV. Conductance values (G) with each voltage pulse (V) were obtained by dividing the peak steady‐state current by the driving force (command voltage minus the measured reversal potential). Conductance‐voltage data were fit by a Boltzmann function, *G* = (*G*_max_ – *G*_min_)/(1 + e^(*V*‐*V½*)/*k*)^) + *G*_min_, where *G*_max_ and *G*_min_ are the maximum and minimum conductance values, *V*_*½*_ the half‐maximal activation voltage, and slope *k*. The liquid junction potential was calculated and was used to correct voltage values after data collection. Inhibition by drugs, which were obtained from commercial sources, was determined from currents evoked by 500 ms voltage ramps from −100 to 0 mV at 0.2 hertz (Hz) as described previously.^16^ Initially, the drugs (10 mM in DMSO) were diluted to 10 μM in a bath solution and applied via gravity perfusion for 2 minutes, followed by at least a 2‐minute wash with drug‐free solution prior to addition of the next drug. Drugs that exhibited inhibition at 10 μM were analyzed further by concentration‐response: *G*/*G*_*C*_ = (1 + ([*B*]/*IC*_*5*0_)^*H*^)^−1^ + *c*, where *G* is the conductance measured as the slope of the current between −60 and 0 mV evoked by the voltage ramp in the presence of the inhibitor, *G*_*C*_ is the control conductance in the absence of inhibitor, [*B*] is the concentration of the inhibitor, *IC*_*50*_ the concentration of inhibitor yielding 50% inhibition, *H* the slope, and *c* the residual conductance.

Inside‐out patch clamp recordings were made at room temperature (23°C) from transiently transfected HEK293T cells, as described previously.^11^ The pipette solution contained, in mM, 140 NaCl, 4 KCl, 2 CaCl_2_, 2 MgCl_2_, and 10 HEPES adjusted to pH 7.4 with NaOH. After achieving the inside‐out configuration, the intracellular face of the membrane was constantly perfused with a solution containing, in mM, 35 NaCl, 110 KCl, 0.2 EGTA, and 10 HEPES adjusted to pH 7.3 with KOH. Drug stock solutions of 5 to 20 mM in DMSO were first diluted in ethanol (1:10) and then in electrophysiological solution. KCNT1 currents were recorded using 2 voltage protocols, depending on the number of active channels in the patch before drug application. Macroscopic currents (number of active channels > 15) were recorded in response to 100 ms voltage ramps between −120 and 120 mV applied every 2 seconds from a holding potential of −78 mV. The average current amplitudes between 0 and 40 mV were used for the analyses and constructing the concentration‐response curves. With a lower number of active channels in the patch (3–15), current traces were recorded in response to 15‐second voltage steps to −20, 0, and 20 mV from a holding potential of −78 mV. Currents recorded at each voltage and each drug concentration were averaged over the duration of the trace and normalized to the average amplitudes of the corresponding current traces in drug‐free solution. The normalized data for all 3 voltage steps were then averaged for each drug concentration and used to build the concentration‐response curves (see Supplementary Fig S3). To determine the IC_50_, data were fitted with the Hill equation, as described previously.^11^ Single channel data were analyzed using Ana software developed by Dr Michael Pusch (Istituto di Biofisica, Genova, Italy; http://users.ge.ibf.cnr.it/pusch/programs-mik.htm). In patch clamp data “n” represents number of cells, and all experiments were repeated using cells from a minimum of 2 separate transfections.

### Drosophila KCNT1 *Epilepsy Models*

The *Drosophila melanogaster* lines carrying the WT human *KCNT1* transgene (NM_020822_3) or *KCNT1* mutant G288S, R398Q or R928C transgene placed downstream of the yeast UAS promoter, have been described previously.^11^ WT or mutant human UAS‐*KCNT1* flies were crossed to flies expressing the GAL4 transcriptional activator under the control of the *GAD1* promoter (GABAergic driver, Bloomington stock number 51630 P2) to drive expression of *KCNT1* in GABAergic neurons, involved in neuronal inhibition.^11^

### *Bang‐Sensitive Behavioral Seizure Assays in* Drosophila

The bang‐sensitive behavioral assay (also known as the banging assay) was used to test for the presence of a seizure phenotype in *Drosophila*.^11^, ^30^ Experiments were performed and scored, as previously described,^11^ with a minimum of 50 *Drosophila* tested for each genotype and drug concentration.

### *Analysis of the Effects of Selected Drugs on Seizure Activity in* Drosophila

Four drugs were selected for in vivo analysis to determine their effects on the seizure phenotypes in transgenic *Drosophila* lines with human mutant *KCNT1* channels. Bepridil, a nonselective KCNT1 inhibitor was included for comparison. *Drosophila* food with and without drugs was prepared as previously described.^11^ All chemicals were obtained from Sigma‐Aldrich (Gillingham, UK) apart from antrafenine hydrochloride (Toronto Research Chemicals, Canada) and regorafenib (USP, Germany).

*Drosophila* crosses were set up, and the resulting embryos were collected, as previously described.^11^ A range of concentrations of each drug dissolved in *Drosophila* food, between 0.001 μM and 1 μM, were used in the experiments. Multiple replicates were performed for each dosage of the drugs and controls. Controls were vehicle controlled (VC), which was the normal food with just the solvent ethanol present (1 μl/1 ml) in the *Drosophila* food, and normal food (NF) control where no drug or solvent (ethanol) was added in the *Drosophila* food.

### *Statistical Analysis of* Drosophila *Studies*

In all experiments, experimenters were not blinded to variants or treatments, nor were samples randomly assigned to groups. Sample sizes were not calculated in advance and no data were excluded from the analysis. Data were analyzed using GraphPad Prism 9 software (San Diego, CA, USA). All values are reported as mean ± standard error of the mean (SEM). All data passed normality and lognormality Shapiro–Wilk tests, determined by GraphPad Prism 9. In the experiments analyzing the effects of drugs in *Drosophila*, statistical significance of differences between groups was determined using Brown‐Forsythe and Welch's analysis of variance (ANOVA) tests (Supplementary Table S3), assuming non‐equal SDs, followed by Dunnett's multiple comparisons tests (GraphPad Prism 9). The results of the Dunnett's multiple comparisons tests are shown in Supplementary Table S4, where “*N*” represents independent trials using independent fly crosses, with the total number of flies for each condition shown in brackets.

### Pharmacokinetics and Brain Uptake of Antrafenine

Plasma pharmacokinetics and brain uptake of antrafenine was analyzed in male C57BL/6 mice. Antrafenine was administered intravenously at 1 mg/kg and 3 mg/kg into the tail vein and plasma was collected over a 24‐hour sampling period (n = 3 mice/time point). Following administration, the brain was harvested at 4 times over 24 hours and snap frozen in dry ice and subsequently stored frozen (−80°C) until analysis. The concentration of antrafenine was determined using liquid chromatography–mass spectrometry. The in vivo study was conducted using established procedures in accordance with the Australian Code of Practice for the Care and Use of Animals for Scientific Purposes, and the study protocols were reviewed and approved by the Monash Institute of Pharmaceutical Sciences Animal Ethics Committee. See Supporting Information for detailed methods and results.

## Results

### Identification and Selection of Drugs for Functional Evaluation

The predicted binding modes of the Drugbank compound library in the KCNT1 intracellular pore vestibule structure were computed and ranked by their docking score. These were further filtered by their clinical status as approved drugs, and manually selected based on their binding mode, commercial availability, and computed hydrophobicity (clogP > 3). Nine drugs were selected for functional assessment: indinavir, antrafenine, candesartan cilexetil, nelfinavir mesylate, regorafenib, dihydrotachysterol, atorvastatin, lifitegrast, and terconazole.

### *Generation of a Cellular Model of Heterozygous Assembly of Wild‐Type and Y796H* KCNT1 *Subunits*

**FIGURE 1:**
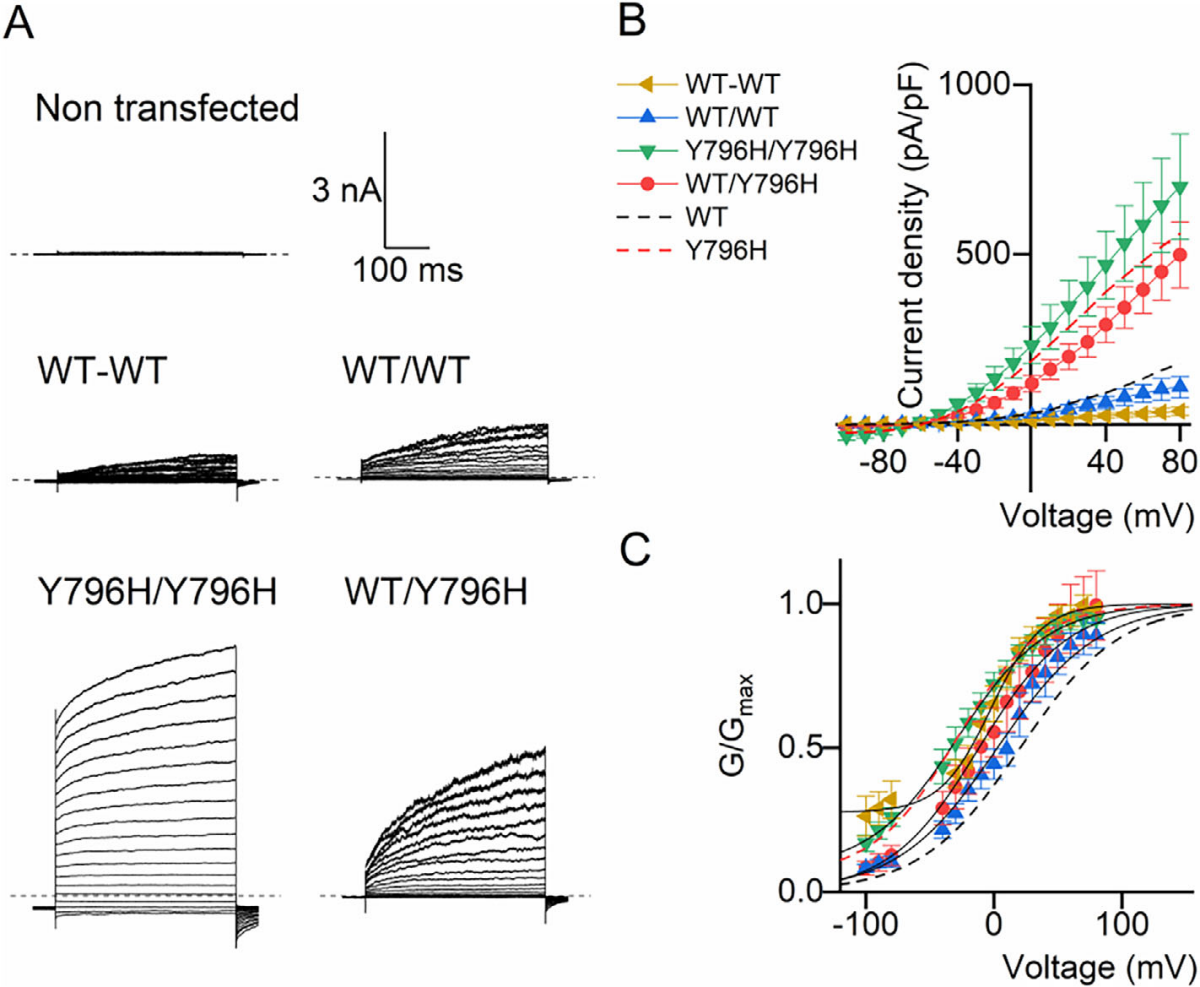
Generation of an expression construct for heteromeric expression of WT and Y796H KCNT1 subunits. The concatemeric constructs encode 2 KCNT1 subunits, separated by either a tethered EGGGSGGGS motif or a T2A self‐cleaving peptide between the first and second subunits. (A) Representative current traces recorded from non‐transfected CHO cells using whole‐cell patch clamp or with CHO cells transfected with constructs containing WT KCNT1 subunits in both the first and second positions (WT‐WT with a hyphen indicates the tethered concatemer; and WT/WT with a slash indicates the cleavable T2A construct), Y796H KCNT1 in both the first and second positions (Y796H/Y796H), or WT in the first and Y796H KCNT1 in the second positions (WT/Y796H), as indicated. The dashed line indicates the zero‐current levels. (B) Current–voltage and (C) conductance‐voltage relationships from the recorded currents. Mean data for monomeric WT KCNT1 (*black*) and Y796H (*red*) are indicated as dashed lines for comparison. Data are mean ± SEM (n = 6 cells for WT‐WT and WT/Y796H; n = 5 cells for WT/WT and Y796H/Y796H). CHO = Chinese hamster ovary; WT = wild‐type. [Color figure can be viewed at www.annalsofneurology.org]

Several KCNT1 channel inhibitors differ in their potency between channels solely comprised of WT subunits or those with a disease‐associated gain‐of‐function mutation.^16^, ^17^, ^31^ Given the heterozygous dominant nature of *KCNT1* disorders, we measured inhibition of channels containing both WT and mutant subunits. Initially, we generated concatemers by fusing 2 WT subunits with a flexible linker. In CHO cells, this yielded outwardly rectifying currents that resembled WT KCNT1 (Fig 1A), but with conductance‐voltage relationships shifted to more negative potentials (Fig 1C). In rodents, different *Kcnt1* mRNA transcripts are generated from alternative transcription initiation sites, leading to variation in the amino terminus of the channel subunit and channels with altered activation kinetics.^32^ We therefore reasoned that constraining the amino terminus of the second subunit in the concatemer may underlie this altered activity and the linker was replaced by a T2A “self‐cleaving” motif. Expression of this construct in CHO cells resulted in similar currents, but with activation kinetics more closely resembling WT channels (see Fig 1). The second subunit in this concatemer was then replaced with one harboring the epilepsy‐causing Y796H mutation. Channels produced from this construct had activation half‐maximal voltages intermediate of homomeric WT and Y796H KCNT1 channels. A summary of the activation kinetics of the monomeric and concatemeric channel currents is provided in Supplementary Table S1.

### *Functional Evaluation of Drugs in Inhibiting WT/Y796H* KCNT1 *Channels*

**FIGURE 2:**
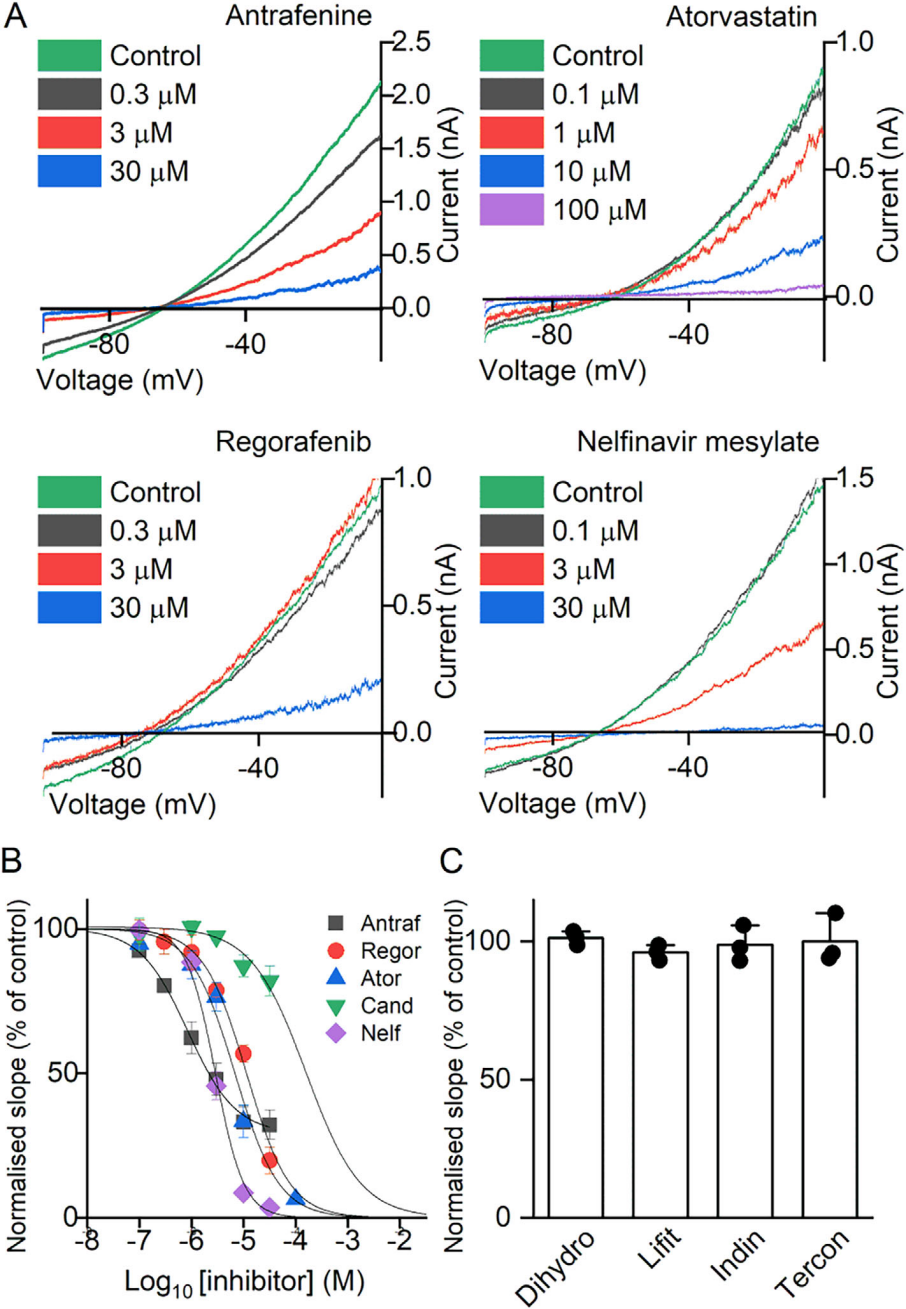
Functional evaluation of drugs with WT/Y796H KCNT1 channels expressed in CHO cells. (A) Representative traces and (B) mean ± SEM. concentration‐dependent inhibition by active inhibitors. Antraf, antrafenine (n = 5 cells); Regor, regorafenib (n = 6 cells); Ator, atorvastatin (n = 6 cells); Cand, candesartan (n = 5 cells); Nelf, nelfinavir (n = 5 cells). (C) Drugs inactive at 10 μM. Data are mean ± SEM WT/Y796H KCNT1 conductance measured as the slope of the current evoked by a voltage ramp to from −100 to 0 mV in the presence of 10 μM inhibitor, relative to control conductance prior to drug application. Dihydro, dihydrotachysterol (n = 3 cells); Lift, lifitegrast (n = 3 cells); Indin, indinavir (n = 3 cells); Tercon, terconazole (n = 3 cells). CHO = Chinese hamster ovary; WT = wild‐type. [Color figure can be viewed at www.annalsofneurology.org]

Using the WT/Y796H KCNT1 construct to model “heterozygous” *KCNT1* pathogenic variants, the selected drugs were evaluated in whole‐cell patch clamp experiments. Four of the drugs, antrafenine, atorvastatin, nelfinavir mesylate, and regorafenib, inhibited the WT/Y796H KCNT1 channels expressed in the CHO cells when tested at 10 μM concentration. Concentration‐inhibition analysis yielded mean ± SEM IC_50_ of 1.30 ± 0.2 μM (n = 5) for antrafenine, 2.86 ± 0.30 μM (n = 5) for nelfinavir mesylate, 7.54 ± 0.99 μM (n = 6) for atorvastatin, and 10.30 ± 1.25 μM (n = 6) for regorafenib (Fig 2A, B). Candesartan cilexetil appeared much less potent (see Fig 2B), and together with indinavir, dihydrotachysterol, lifitegrast, and terconazole, which did not exhibit inhibition at 10 μM concentration (Fig 2C), they were not studied any further.

**FIGURE 3:**
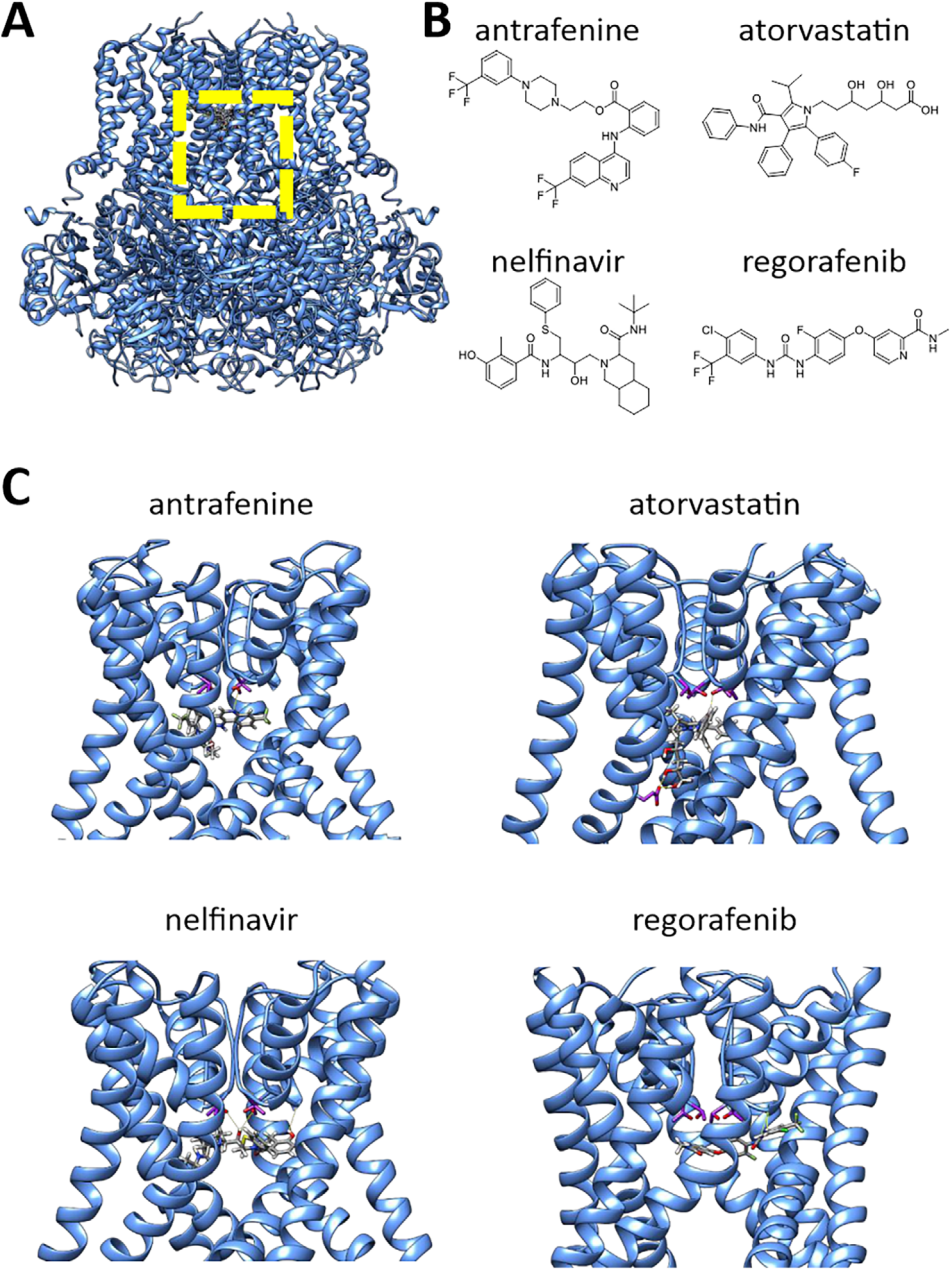
Molecular docking of drugs to the KCNT1 channel pore. (A) Structure of chicken KCNT1 in the active conformation (PDB: 5U70)^24^ with drugs docked to the channel pore domain, indicated by the yellow dashed box. (B) Molecular structures of active drugs identified in this study. (C) Individual active drugs docked to the KCNT1 pore domain as indicated. For clarity, only the S5, pore helix, selectivity filter, and S6 of each subunit is shown and rotated to best illustrate each drug binding mode. Threonine sidechains at the intracellular end of the selectivity filter are colored magenta, also the aspartate side chain in the S6 segment that interacts with atorvastatin. [Color figure can be viewed at www.annalsofneurology.org]

Similar to the inhibitors that we previously identified by virtual screening,^16^ these drugs were predicted to bind to the intracellular vestibule of the KCNT1 channel pore below the selectivity filter (Fig 3 and Supplementary Fig S1). Each are predicted to make hydrophobic and hydrogen bond interactions with pore‐lining amino acids, here provided as the equivalent amino acid and number in the human KCNT1 homolog. Antrafenine and atorvastatin are both predicted to make a hydrogen bonding interaction with the side chain of T314, which forms the intracellular‐facing part of the selectivity filter, with atorvastatin making 2 additional hydrogen bonding interactions with the E347 side chain in the S6 segment. Nelfinavir and regorafenib are both predicted to hydrogen bond with the backbone carbonyl of F312, with nelfinavir also interacting with the side chain of T314. Antrafenine contains 2 and regorafenib contains 1 terminal trifluoromethyl group. Similar to the predicted binding modes of BC12 and BC14,^16^ we found that these terminal groups occupy a cavity formed between adjacent S6 segments and the pore helix. Antrafenine spans the pore, with the 2 trifluoromethyl groups at the opposite ends of the molecule occupying the equivalent cavities formed by opposite subunits in the KCNT1 tetramer. Since carrying out this docking, structures of human KCNT1 in several states, including with an inhibitor bound, were published.^33^ With the exception of atorvastatin, there is good agreement in the docking scores of these drugs between the chicken and inhibitor‐bound human KCNT1 structures (Supplementary Table S2), with close conservation of both sequence and structure in the pore domain (Supplementary Fig S2).

### *Inhibition of Single Human* KCNT1 *Channels by the Four Prioritized Drugs*

**FIGURE 4:**
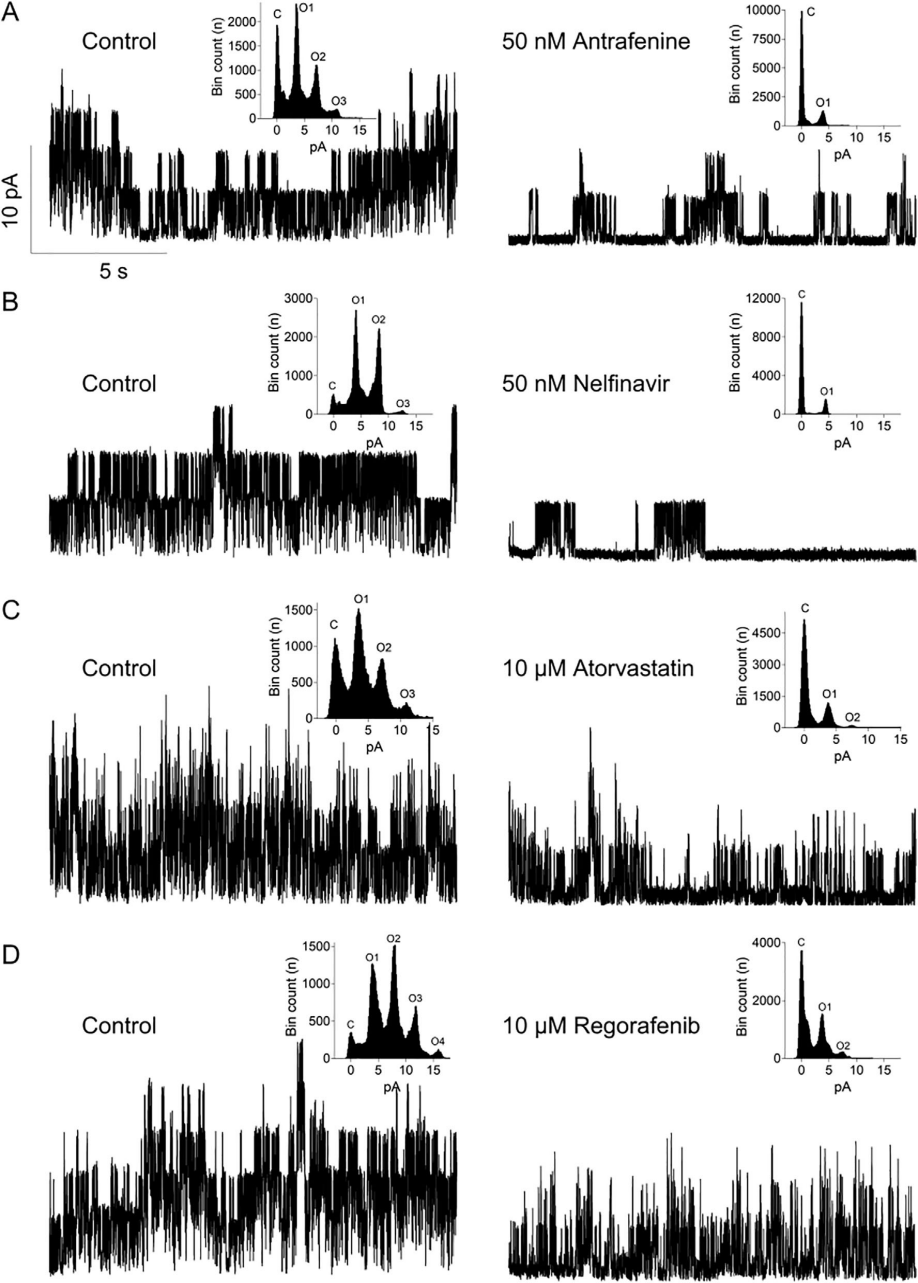
Functional assessment of drug efficacy using inside‐out patch clamping of HEK293T cells expressing WT KCNT1 channels. Single channel current traces were recorded at 0 mV and physiological K^+^ gradient under control conditions and in the presence of different drugs: (A) antrafenine (50 nM), (B) nelfinavir mesylate (50 nM), (C) atorvastatin (10 μM), and (D) regorafenib (10 μM). Insets show all‐point amplitude histograms of the corresponding traces (C closed and O open levels). WT = wild‐type.

**FIGURE 5:**
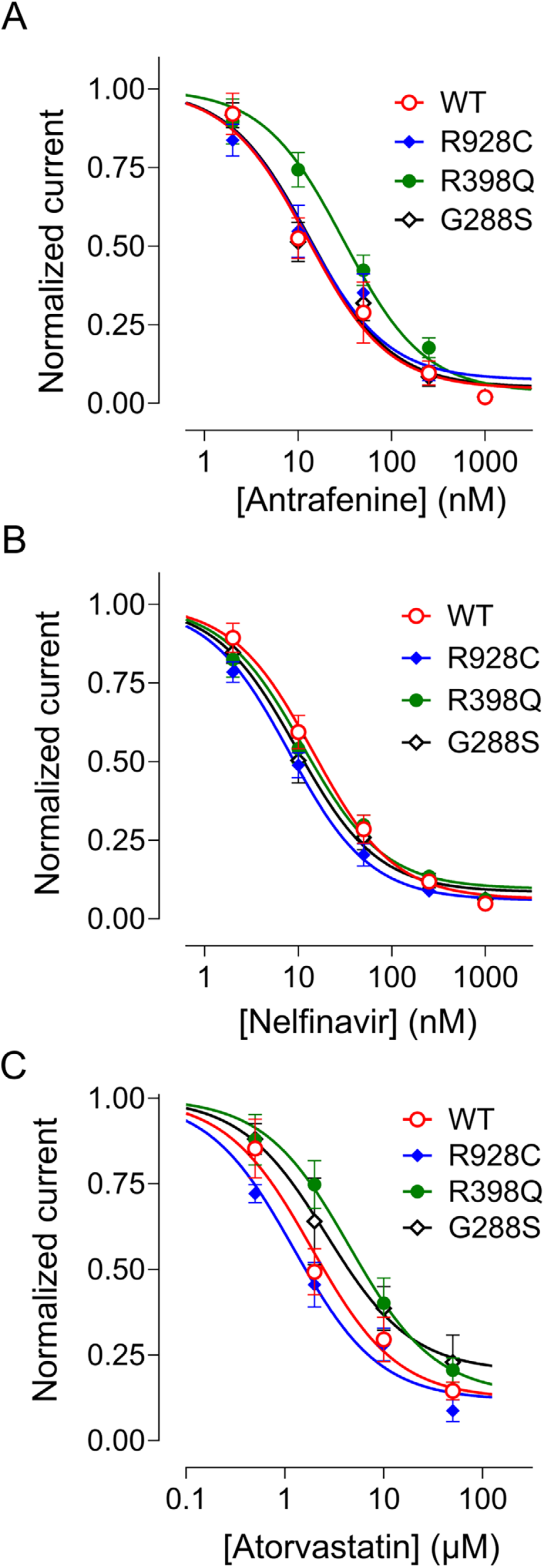
Assessment of known drug efficacy in inhibiting KCNT1 channels carrying epilepsy‐causing mutations, G288S, R398Q, and R928C. Concentration‐response curves were constructed using KCNT1 currents (see Supplementary Fig S3) recorded in inside‐out patches in the presence of different concentrations of a drug in the bath and normalized to the current amplitude recorded before drug application. The data points were fitted with a Hill equation with a slope of −1. (A) Antrafenine, (B) nelfinavir, and (C) atorvastatin. (For *IC*_*50*_ values see the Table 1.) WT = wild‐type. [Color figure can be viewed at www.annalsofneurology.org]

Antrafenine, nelfinavir mesylate, atorvastatin, and regorafenib were further analyzed by investigating their effects on unitary WT and R928C, G288S, and R398Q mutant KCNT1 channels expressed in HEK293T cells in inside‐out patches. The tight seal between the patch pipette and the cell was achieved in the control bath solution, after which the inside‐out patch was excised and moved under the outlet of the gravity‐fed perfusion system used to apply drugs to the intracellular face. Antrafenine and nelfinavir inhibited over 60% WT human KCNT1 channel activity at concentrations of 50 nM (Fig 4A, B). The effects of atorvastatin and regorafenib on KCNT1 channels were less potent, with the drugs not fully inhibiting KCNT1 activity at concentrations of 10 μM (Fig 4C, D). Each drug reduced the open probability of KCNT1 without affecting the single channel conductance (see Fig 4 insets). Antrafenine, nelfinavir mesylate, and atorvastatin had similar inhibitory effects on the WT and each of the R928C, G288S, and R398Q mutant KCNT1 channels at the same concentrations (see Fig 5, see the Table 1, and Supplementary Fig S3).

**TABLE 1:** Potency of Drugs Inhibiting WT and Mutant KCNT1 Channels in Inside‐Out Patches

Variant	Antrafenine	Nelfinavir	Atorvastatin
WT	12.6 ± 3.6 nM (n = 6)	14.3 ± 2.6 nM (n = 6)	1.9 ± 0.6 μM (n = 5)
R928C	12.3 ± 3.9 nM (n = 6)	8.0 ± 1.1 nM (n = 5)	1.2 ± 0.3 μM (n = 4)
R398Q	30.7 ± 7.4 nM (n = 5)	11.2 ± 2.2 nM (n = 6)	4.5 ± 1.7 μM (n = 5)
G288S	13.3 ± 3.7 nM (n = 5)	9.5 ± 1.8 nM (n = 6)	2.6 ± 1.6 μM (n = 3)

*IC*_*50*_ values for antrafenine, nelfinavir, and atorvastatin‐mediated inhibition of WT and mutant KCNT1 channels in inside‐out patches, derived from concentration‐response curves shown in Figure 5. Data are mean ± SEM of n patches.

WT = wild‐type.

### *In Vivo Analysis of the Four Prioritized Drugs Fed to* Drosophila *Models of* KCNT1 *Epilepsy*

To analyze the effects of the 4 prioritized drugs on *KCNT1*‐related seizures in vivo, we used our humanized *Drosophila* models of *KCNT1* epilepsy. The models contain human *KCNT1* with patient‐specific mutations G288S, R398Q, or R928C. *KCNT1* expression was under the control of the yeast UAS promoter and crossing to a line with the GAL4 transcription factor under the control of a chosen promoter, which enables tissue‐specific expression of the transgene.^34^ We have shown previously that expression of mutant KCNT1 channels in GABAergic inhibitory neurons gives a seizure phenotype in bang‐sensitive assays and that these models can be used to analyze the effects of *KCNT1*‐inhibiting drugs.^11^

**FIGURE 6:**
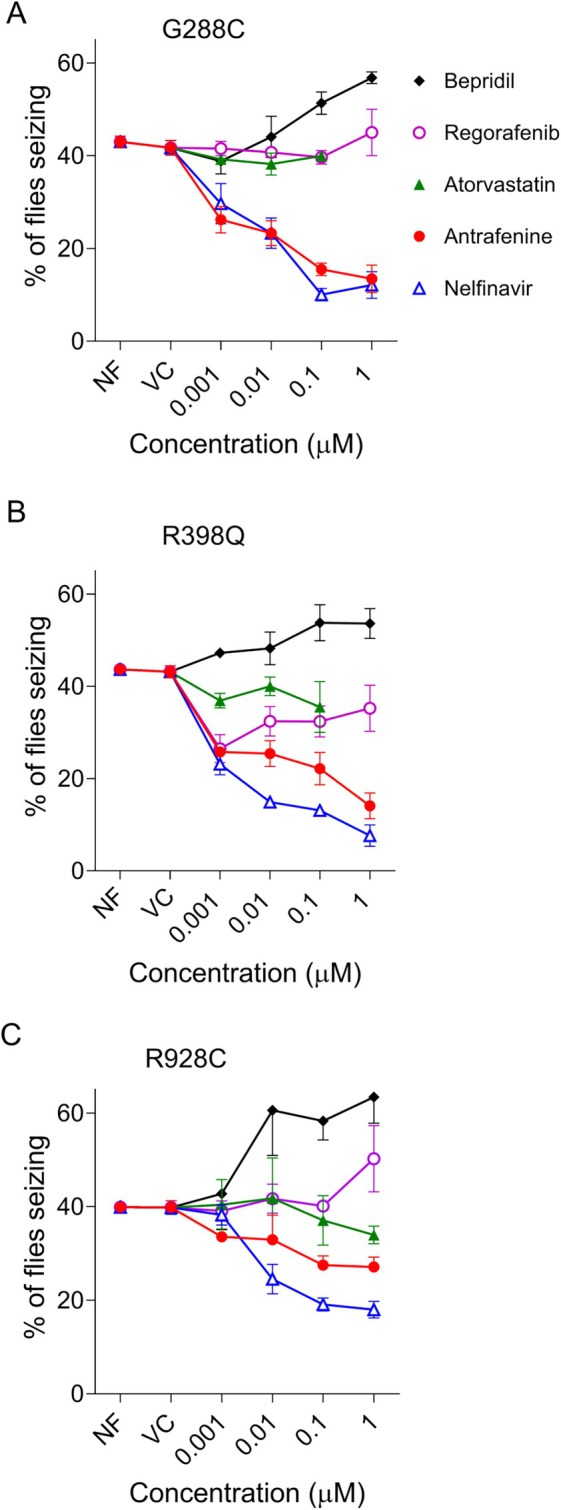
Reduction of seizure phenotype in three *Drosophila KCNT1* mutant lines by known drugs. The percentage of adult *Drosophila* showing seizure activity is shown for each of the *KCNT1* mutant lines G288S, R398Q, or R928C when raised on NF or food with added VC, or the known drugs nelfinavir mesylate, antrafenine, atorvastatin, or regorafenib. Seizures in *Drosophila* are caused by expression of human *KCNT1* transgenes with mutations in the pore‐adjacent loop domain, G288S (A), RCK1 domain, R398Q (B), or the RCK2 domain, R928C (C), in inhibitory GABAergic neurons. Data are presented as mean ± SEM. NF = normal food; VC = vehicle control. [Color figure can be viewed at www.annalsofneurology.org]

Juvenile *Drosophila* (larvae) from the lines expressing G288S, R398Q, or R928C, human *KCNT1*, were fed each of the drugs and adult *Drosophila* were analyzed for seizure activity using bang‐sensitive assays. Each mutant line was also raised on normal *Drosophila* food (NF) or normal *Drosophila* food containing the vehicle ethanol (VC), which was used to introduce each of the drugs to the *Drosophila* food. These 2 controls showed the baseline seizure activity of each of the mutant *KCNT1 Drosophila* lines (Fig 6). As shown in Figure 2, 6, 2 of the drugs, antrafenine and nelfinavir mesylate, were seen to significantly reduce the seizure activity at concentrations 1 nM to 1 μM (inclusive) in each of the three *Drosophila* mutant *KCNT1* lines in a dose dependent manner (see Supplementary Tables S3 and S4 for statistics). The other 2 drugs, atorvastatin and regorafenib, did not show a significant effect on seizure activity in the G288S, R398Q, or R928C mutant lines. Analysis of 1 μM atorvastatin was excluded due to reduced viability of adult flies at this concentration. The nonspecific inhibitor, bepridil, was included for comparison and seen to exacerbate the seizure phenotype in each of the 3 *KCNT1* mutant lines (see Fig 6) as was observed with quinidine.^11^ Nelfinavir mesylate showed the greatest reduction in seizure activity, followed by antrafenine. Previous publications on variable pharmacokinetics, drug–drug interactions, and limited brain uptake,^35^, ^36^, ^37^ raised concerns of the suitability of nelfinavir as a potential anti‐epilepsy drug and it was not further investigated in this study.

### Investigation of Blood–Brain Barrier Penetration of Antrafenine in the Mouse

To further investigate the potential suitability of antrafenine as a drug for epilepsy, its ability to cross the blood brain barrier was analyzed in mice. Plasma pharmacokinetics and commensurate brain uptake of antrafenine were determined by liquid chromatography‐mass spectrometry after intravenous administration of 3 mg/kg antrafenine in C57BL/6 mice. The half‐life of antrafenine in plasma was found to be 7.4 hours (Supplementary Table S6). At 4 hours after administration, the concentration of antrafenine in the brain was approximately 30% of that present in plasma, with a mean plasma concentration of 47.5 nM (+5.9 nM SD) and a mean brain concentration of 13.9 nM (+3 nM SD; Supplementary Table S7).

## Discussion

We have successfully used in silico screening to identify 4 existing drugs that inhibit KCNT1 channels at low micromolar potency in whole‐cell patch clamp recordings, and importantly more potently than the known KCNT1 inhibitor quinidine. The outcomes from this approach are similar to those we obtained previously with a library of commercially available small molecules.^16^ Because the 4 drugs have already been considered for therapeutic use, pharmacological and clinical data are available to inform whether they can be trialed for treating *KCNT1*‐associated disorders. Using the inside‐out patch clamp recordings, antrafenine and nelfinavir were seen to inhibit 80 to 90% of the activity of the WT and mutant human KCNT1 channels at 250 nM. This enhanced sensitivity, compared to the whole‐cell patch clamp, supports the idea that the drugs block the channel at the intracellular pore vestibule of the channel, either via the cytoplasm or directly from the lipid bilayer.

Based on the in vitro analysis and their inhibition of KCNT1 channels, the 4 drugs antrafenine, atorvastatin, nelfinavir mesylate, and regorafenib, were selected to be analyzed in vivo for their effects on the seizure phenotype in 3 humanized *Drosophila* models of *KCNT1* epilepsy. *Drosophila* was chosen as an animal model as the key components in the regulation of neuronal excitability in humans and *Drosophila* are highly conserved.^38^ Other *Drosophila* models of human genetic epilepsies include those for sodium channel *SCN1A*‐related epilepsy^39^ and Pyridox(am)ine 5′‐phosphate oxidase (*PNPO*)‐related epilepsy.^40^ The relative ease of genetic manipulation, fast generation times, and the low cost of housing make *Drosophila* attractive for modeling human epilepsy and for relatively rapid drug analyses.

The normal role of the KCNT1 channel is to reduce neuronal excitability and so it appears paradoxical that mutations leading to overactivity of the channel could contribute to neuronal hyperexcitability in epilepsy. However, this may be explained by accumulating evidence that overactivity of mutant KCNT1 channels in inhibitory neurons contributes to pathogenicity in *KCNT1* epilepsy.^7^, ^8^, ^9^, ^10^, ^11^, ^12^ Our *Drosophila* models expressed mutant human KCNT1 channels in inhibitory GABAergic neurons, exhibited a seizure phenotype, and were used for screening drugs for the ability to suppress seizures. *KCNT1* is widely expressed in the human brain (The Human Brain Atlas). However, expression of mutant KCNT1 channels in all neurons is embryonic lethal in *Drosophila*,^11^ therefore, it is unknown if widespread expression of mutant KCNT1 channels would influence the seizure phenotype. Feeding of antrafenine or nelfinavir mesylate were found to significantly reduce the seizure phenotype in each *Drosophila* mutant *KCNT1* line, whereas atorvastatin and regorafenib did not. Nelfinavir was seen to reduce the seizure phenotype by 50% in *Drosophila* with G288S and R398Q and R928C. Antrafenine reduced the phenotype by at least 50% in G288S and R398Q, with a 25% reduction in R928C. The reduction of the seizure phenotype was found to be dose‐dependent for each of these drugs, showing suppression at 0.001 μM, with the greatest effects at 0.1 μM or 1 μM in food. Based on physiological effective concentrations, the recommendation for drug screening in *Drosophila* is at concentrations between 0.1 μM and 10 μM added to the feeding substrate.^41^ Antrafenine and nelfinavir were found to suppress seizures in the *KCNT1* mutant *Drosophila* lines at concentrations between 0.001 μM and 1.0 μM μM.^42^ This suggests that the KCNT1 channel‐blocking activities of antrafenine and nelfinavir are within an appropriate potential therapeutic range. Phenytoin, lamotrigine, and valproate were seen to reduce seizures by approximately 30% in the *Drosophila* epilepsy model.^42^ The reduction of seizures by approximately 25 to 75% by antrafenine and nelfinavir in this study support their consideration as new drugs for epilepsy.

The 2 potential new drugs for treating people with *KCNT1* epilepsy, antrafenine and nelfinavir mesylate, are yet to be trialed in patients for this indication. Nonselective cation channel blocking drugs, such as quinidine and bepridil, have been shown to inhibit KCNT1 channels in in vitro experiments,^31^, ^43^ with quinidine having been trialed as a stratified treatment for *KCNT1* epilepsy.^44^, ^45^, ^46^, ^47^ However, quinidine has had mixed results in treating patients with worsening of seizures in some of the patients.^1^, ^48^ The use of bepridil would be limited in clinical settings over safety concerns and was withdrawn for this reason.^49^ We have shown that both bepridil (this study) and quinidine^11^ exacerbate the seizure phenotype in our *Drosophila* models of *KCNT1* epilepsy, most likely by inhibiting other cation channels that control neuronal excitability, highlighting the value of in vivo preclinical analysis for drug repurposing. Interestingly, seizures were most exacerbated by bepridil in the R928C mutant line, suggesting mutation‐specific differences in response to drugs and indicating a role for mutation‐specific pharmacogenomics.

Nelfinavir mesylate and antrafenine showed significant reduction in seizure activity associated with all 3 *KCNT1* patient mutations R928C, R398Q, and G288S when tested in our animal models. They also showed significant reduction of K^+^ currents in HEK cells expressing the same 3 mutant channels, as well as channels comprised of WT and Y796H mutant KCNT1 subunits in CHO cells. These data suggest that these 2 drugs have the potential to inhibit a spectrum of mutant KCNT1 channels found in patients and, if found to be clinically effective, could be new treatments for patients with a range of different *KCNT1* mutations. Apart from antrafenine, each of the KCNT1‐inhibiting drugs described here are presently in clinical use. Atorvastatin (brand name Lipitor) is a competitive inhibitor of 3‐hydroxy‐3‐methyl‐glutaryl‐coenzyme A (HMG‐CoA) reductase, an enzyme involved in cholesterol synthesis, and is a statin used in the treatment of hyperlipidemia and hypercholesterolemia. Partial inhibition of hERG currents by atorvastatin at low micromolar concentrations have been reported and appear to affect hERG channel inactivation kinetics.^50^ Regorafenib (brand name Stivarga) is an anticancer drug that targets receptor tyrosine kinases and is presently under clinical evaluation for treating brain tumors.^51^ Of the drugs with efficacy in *Drosophila*, nelfinavir mesylate (brand name Viracept) is an HIV‐1 protease inhibitor. It has been reported that this drug inhibits hERG potassium channels with an IC_50_ of 11.5 μM,^52^ which could place patients at risk of arrhythmia. Its CNS penetrance is believed to be limited by its extrusion by P‐glycoprotein in the blood–brain barrier,^53^ which may prevent concentrations reaching levels required to inhibit KCNT1. Antrafenine (brand name Stakane) has analgesic and anti‐inflammatory effects, but there is no record of its clinical availability or use since the mid‐1980s. Pharmacokinetic studies of this drug are lacking, although the QPlogBB score (see Supplementary Table S2) is within a range of drugs that cross the blood–brain barrier. In a clinical study with patients with osteoarthritis, mean circulating plasma levels of 0.3 μM antrafenine were measured,^54^ which is in the concentration range of the KCNT1 channel inhibition reported here. We evaluated the pharmacokinetic properties of brain uptake of antrafenine in the mouse and found significant penetration of antrafenine into the brain at concentrations close to 30% of those present in plasma. No toxic effects of antrafenine in mice were noted in this study, consistent with previous studies in mice and rats,^55^ and clinical studies have shown that the drug is well tolerated in humans at 900 mg/day.^54^ Based on these findings, we consider antrafenine as the lead candidate new drug for *KCNT1* epilepsy from this study. Seizure reduction efficacy in rodent models of *KCNT1* epilepsy will be required to justify its future clinical evaluation in patients with *KCNT1* epilepsy.

## Author contributions

**Michael G. Ricos:** Investigation; writing – review and editing; formal analysis. **Bethan A. Cole:** Investigation; formal analysis. **Rashid Hussain:** Investigation; formal analysis. **Grigori Y. Rychkov:** Investigation; formal analysis; writing – review and editing. **Zeeshan Shaukat:** Investigation; formal analysis. **Nadia Pilati:** Supervision; writing – review and editing; funding acquisition. **Stephen P. Muench:** Formal analysis; writing – review and editing. **Katie J. Simmons:** Writing – review and editing; formal analysis; investigation. **Leanne M. Dibbens:** Conceptualization; writing – original draft; funding acquisition; supervision. **Jonathan D. Lippiat:** Writing – original draft; supervision; conceptualization; funding acquisition.

## Potential Conflicts of Interest

The authors declare that they have no conflicts of interest.

## Supporting information


**Data S1.** Supporting information.

## Data Availability

The data that support the findings of this study are available from the corresponding authors, upon reasonable request.
