# Supplementary material for: Identification of New KCNT1‐Epilepsy Drugs by In Silico, Cell, and Drosophila Modeling
Source: Ann Neurol. 2025 Sep 13;98(6):1261–74. doi: 10.1002/ana.78031 (PMC12682947; doi:10.1002/ana.78031)

Identification of new KCNT1-epilepsy drugs by *in silico,* cell and *Drosophila* modelling

Supplementary Results and Methods

Supplementary Table 1: Parameters derived from Boltzmann analysis of WT, Y796H, and concatemeric K_{Na}1.1 currents.

Data are the mean ± SEM (of *n* number of cells provided in 5th column) half-maximal activation voltages ($V_{½}$) and apparent gating charge (*z*, obtained from the slope $k = RT/zF$) the Boltzmann function fitted to conductance-voltage plots. Currents were recorded from CHO cells transiently transfected with plasmids to form homotetrameric WT or Y796H K_{Na}1.1 channels, or with concatemeric homomeric or heteromeric constructs. A hypthen "-" between the subunits indicates the tethered tandem dimer (linker: GGGSGGGS) and the forward slash (/) between the subunits indicates that they are linked by the cleavable T2A motif (linker: GSGEGRGSLLTCGDVEENPG).

KCNT1 construct	Linker	$V_{½}$ (mV)	*z*	*n*
WT	none	7.38 ± 4.35	1.01 ±0.13	6
WT-WT	Tethered dimer	-15.72 ± 1.85	1.46 ±0.17	5
WT/WT	Cleavable T2A	-2.74 ± 7.69	0.89 ±0.07	5
Y796H	none	-39.99 ± 2.27	0.92 ±0.08	6
Y796H-Y796H	Tethered dimer	-50.83 ± 5.26	0.92 ±0.16	5
Y796H/Y796H	Cleavable T2A	-42.19 ± 7.97	0.82 ±0.12	5
WT/Y796H	Cleavable T2A	-16.42 ± 8.97	0.71 ±0.09	6
Y796H/WT	Cleavable T2A	-21.51 ± 5.86	0.80 ±0.08	6

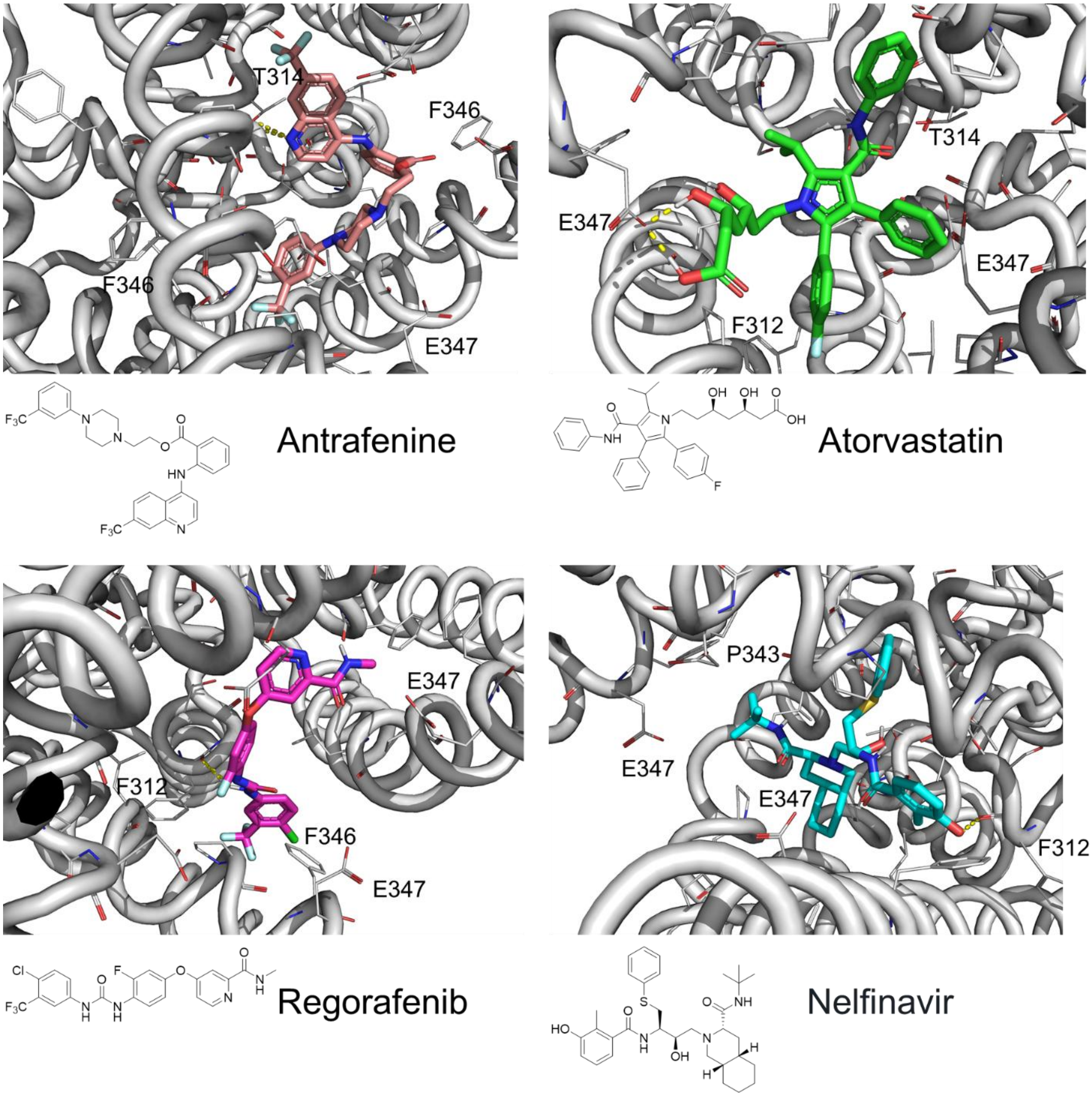

Supplementary Figure 1: Predicted interactions of drugs with the K_{Na}1.1 pore domain (PDB: 5U70) though molecular docking in Glide (Schrödinger).

Supplementary Table 2. Computationally-derived predicted properties of drugs using Schrödinger software. Glide docking scores for drugs interacting with the chicken activated KCNT1 channel structure (PDB: 5U70) and the inhibitor-bound human KCNT1 channel structure (PDB:8HKQ), and QikProp predictions of blood-brain-barrier (BBB) permeability (QPlogBB) and overall CNS activity (CNS). Docking scores that are more negative predict a higher affinity. QPlogBB values are the predicted logarithm of BBB partition coefficient, with positive values consistent with drugs that have good penetrance. CNS scores are between -2 (likely CNS inactive) to 2 (likely CNS active).

	**Glide docking scores**		**QikProp values**	
**Drug**	**Chicken**	**Human**	**QPlogBB**	**CNS**
Antrafenine	-8.88	-8.25	0.248	1
Atorvastatin	-7.59	-5.90	-2.092	-2
Nelfinavir	-8.01	-8.22	-0.849	-1
Regorafenib	-7.88	-8.32	-0.933	-1

A

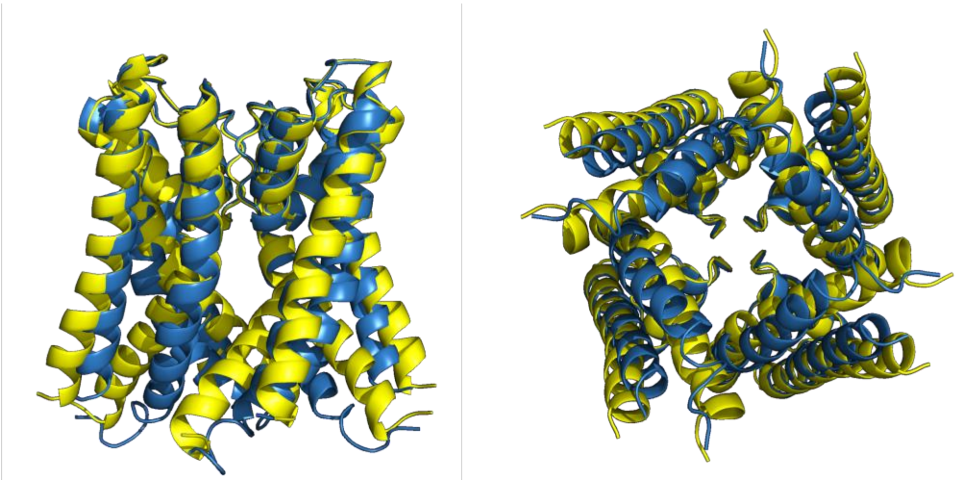

B

<-------------S5------------>   <-----PH----->

Gga ^{244}SAMFNQVLILICTLLCLVFTGTCGIQHLERAGEKLSLFKSFYFCIVTFS

Hsa ^{265}SAMFNQVLILFCTLLCLVFTGTCGIQHLERAGENLSLLTSFYFCIVTFS

<-SF->   <--------------S6------------->

Gga TVGYGDVTPKIWPSQLLVVIMICVALVVLPLQFEELVYLWMERQKSGG^{340}

Has TVGYGDVTPKIWPSQLLVVIMICVALVVLPLQFEELVYLWMERQKSGG^{361}

Supplementary Figure 2. Conservation in the pore domain between the chicken and human KCNT1 structures. A Three-dimensional alignment of pore domains from the structures of chicken (yellow, PDB:5U70) and human (blue, PDB:8HIR) active KCNT1. The root mean square deviation between atoms is 1.3 Å. **B** Sequence alignment of the chicken (*Gga*) and human (*Hsa*) KCNT1 pore domains indicated in **A** comprising the S5 and S6 transmembrane segments, the pore helix (PH), and selectivity filter (SF) as indicated. Differences between species are highlighted in yellow.

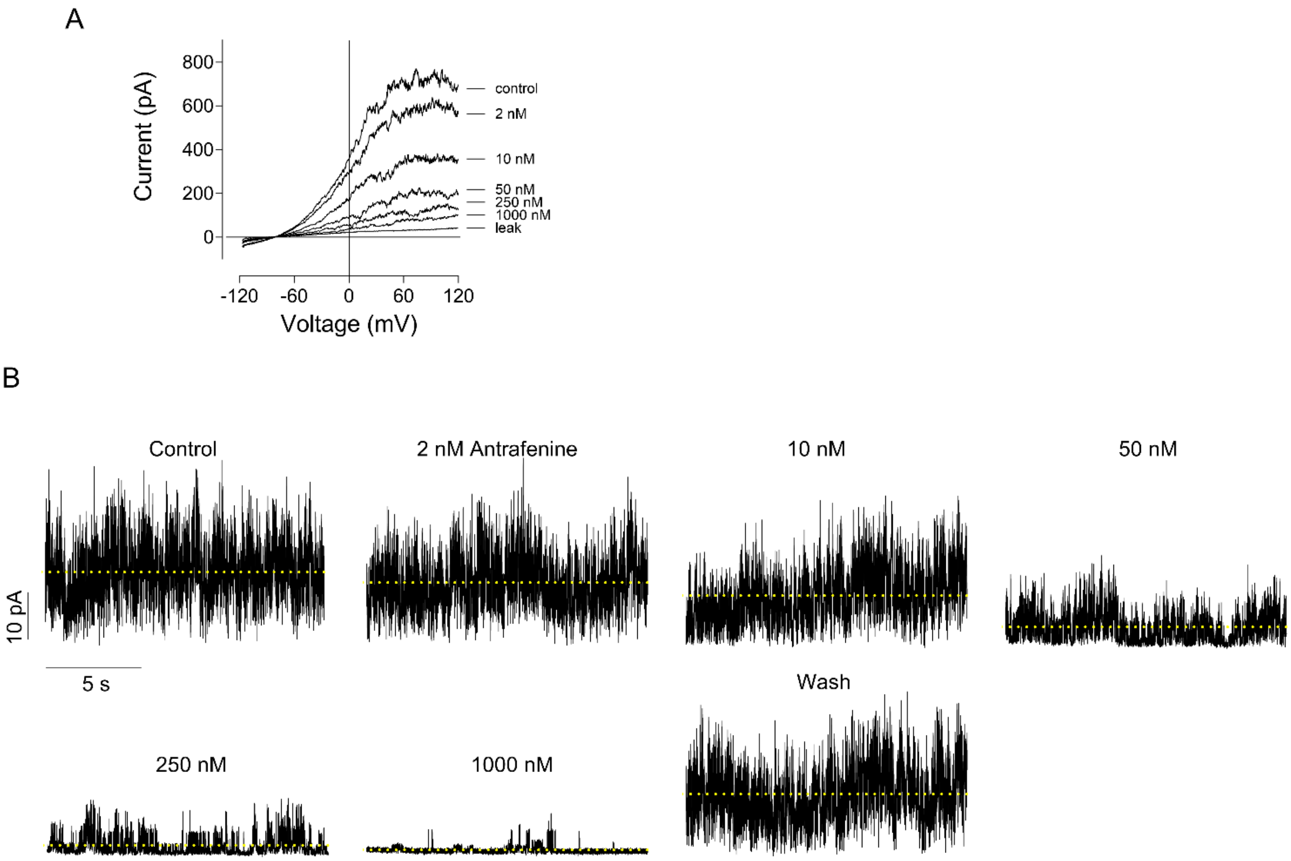

Supplementary Figure 3. Examples of the current traces used to build concentration-response curves. A Macroscopic G288S KCNT1 currents recorded in the inside-out patch in the presence of different concentrations of nelfinavir. Leak current was determined by replacing KCl in the bath solution with equimolar amount of NaCl. **B** Recording of a current in an inside-out patch containing 10 – 15 R398Q KCNT1 channels in the presence of different concentrations of Antrafenine, at 0 mV. The dashed lines represent the mean current amplitude of each trace.

Supplementary Table 3. One-way ANOVA results from the analysis of the Drosophila seizure phenotype data presented in Figure 6. ns - not significant.

**G288S**					
	Antrafenine	Nelfinavir	Atorvastatin	Regorafenib	Bepridil
F	44.78	52.39	1.529	0.7738	7.947
P value	<0.0001	<0.0001	0.2317	0.5799	0.0002
P summary	****	****	ns	ns	***
R squared	0.8924	0.8881	0.2342	0.1621	0.6234

**R398Q**					
	Antrafenine	Nelfinavir	Atorvastatin	Regorafenib	Bepridil
F	25.10	68.90	3.880	8.160	4.379
P value	<0.0001	<0.0001	0.0192	0.0002	0.0069
P summary	****	****	*	***	**
R squared	0.8451	0.9323	0.4630	0.6602	0.5104

**R928C**					
	Antrafenine	Nelfinavir	Atorvastatin	Regorafenib	Bepridil
F	5.341	35.92	0.8976	1.970	8.246
P value	0.0001	<0.0001	0.4932	0.1076	<0.0001
P summary	***	****	ns	ns	****
R squared	0.3022	0.7623	0.1108	0.2196	0.5076

Supplementary Table 4. P values obtained using One way ANOVA with Dunnett's multiple comparisons test of the data presented in Figure 6. Blue asterisks denote the statistically significant decrease in seizures compared to vehicle control, whereas red asterisks denote significant increase. ns – not significant. N is the number of independent experiments; number in brackets is the total number of flies analysed in each condition. For the controls, the number of independent experiments and the total number of flies for G288S, R398Q and R928C are 8 (88), 7 (141) and 12 (140), respectively.

	**G288S**					**R398Q**					**R928C**				
**[μM]**	**Antraf**	**Nelf**	**Atorv**	**Regor**	**Bepr**	**Antraf**	**Nelf**	**Atorv**	**Regor**	**Bepr**	**Antraf**	**Nelf**	**Atorv**	**Regor**	**Bepr**
**0.001**	<0.0001<br>****<br>N=4<br>(79)	0.0052<br>**<br>N=4<br>(54)	0.3267<br>ns<br>N=4<br>(136)	>0.9999<br>ns<br>N=2<br>(108)	0.5514<br>ns<br>N=4<br>(85)	0.0003<br>***<br>N=3<br>(62)	<0.0001<br>****<br>N=5<br>(261)	0.0359<br>***<br>N=5<br>(113)	0.0002<br>***<br>N=4<br>(135)	0.5430<br>ns<br>N=4<br>(95)	0.6765<br>ns<br>N=4<br>(54)	0.9534<br>ns<br>N=12<br>(170)	0.9998<br>ns<br>N=4<br>(59)	0.9997<br>ns<br>N=5<br>(56)	0.9923<br>ns<br>N=4<br>(46)
**0.01**	<0.0001<br>****<br>N=5<br>(143)	<0.0001<br>****<br>N=5<br>(71)	0.1480<br>ns<br>N=4<br>(114)	0.9929<br>ns<br>N=4<br>(54)	0.9968<br>ns<br>N=4<br>(78)	<0.0001<br>****<br>N=6<br>(126)	<0.0001<br>****<br>N=4<br>(119)	0.5236<br>ns<br>N=4<br>(122)	0.0206<br>*<br>N=4<br>(57)	0.3394<br>ns<br>N=4<br>(74)	0.4387<br>ns<br>N=6<br>(102)	<0.0001<br>****<br>N=6<br>(115)	0.9936<br>ns<br>N=4<br>(67)	0.9865<br>ns<br>N=5<br>(56)	0.0296<br>*<br>N=3<br>(57)
**0.1**	<0.0001<br>****<br>N=5<br>(101)	<0.0001<br>****<br>N=10<br>(153)	0.7150<br>ns<br>N=2<br>(103)	0.9009<br>ns<br>N=3<br>(145)	0.0781<br>ns<br>N=3<br>(78)	<0.0001<br>****<br>N=5<br>(144)	<0.0001<br>****<br>N=2<br>(77)	0.0676<br>ns<br>N=2<br>(89)	0.0386<br>*<br>N=3<br>(175)	0.0152<br>*<br>N=3<br>(66)	0.0041<br>**<br>N=13<br>(216)	<0.0001<br>****<br>N=11<br>(177)	0.9720<br>ns<br>N=4<br>(64)	>0.9999<br>ns<br>N=4<br>(55)	0.0049<br>**<br>N=8<br>(110)
**1**	<0.0001<br>****<br>N=4<br>(56)	<0.0001<br>****<br>N=5<br>(73)		0.7301<br>ns<br>N=3<br>(64)	0.0005<br>***<br>N=4<br>(65)	<0.0001<br>****<br>N=3<br>(71)	<0.0001<br>****<br>N=8<br>(113)		0.1793<br>ns<br>N=3<br>(70)	0.0080<br>**<br>N=4<br>(55)	0.0006<br>***<br>N=24<br>(301)	<0.0001<br>****<br>N=11<br>(161)	0.3850<br>ns<br>N=8<br>(118)	0.0360<br>*<br>N=5<br>(63)	0.0002<br>***<br>N=9<br>(140)

PHARMACOKINETICS AND BRAIN UPTAKE OF ANTRAFENINE

Plasma pharmacokinetics and brain uptake of antrafenine was analyzed in male C57BL/6 mice by the Monash University Centre for Drug Candidate Optimisation. Antrafenine was administered intravenously at 1 mg/kg and 3 mg/kg into the tail vein and plasma was collected over a 24 h sampling period (n=3 mice/time point). Following administration, brain was harvested at four times over 24 hours and snap frozen in dry ice and subsequently stored frozen (-80°C) until analysis. The concentration of antrafenine was determined using liquid chromatography–mass spectrometry (LC-MS).

Summary of findings

The half-life of a 3 mg/kg IV administration of antrafenine in plasma was 7.4 hours. Given this, 4 hrs post IV administration of 3 mg/kg Antrafenine was taken as a representative time to compare plasma and brain concentrations. At 4 hrs we determined a mean plasma concentration of 47.5 nM (±5.9 nM SD) and a mean brain concentration of 13.9 nM (±3 nM SD). This showed significant penetration of Antrafenine into the brain at concentrations close to 30% of those present in plasma. This is clinically significant as the direct IC_{50} for antrafenine on single KCNT1 channels in inside-out patches was in the 10 nM range with an almost 90% inhibition at 300 nM. Historically, studies have shown that administering 900 mg/day which is approximately 12 mg/kg for a 75 kg adult resulted in a mean plasma concertation of 202 nM (Berry *et al*, 1983). As such, administration of Antrafenine should reach therapeutic concentrations higher than this IC_{50}.

Methods

Plasma pharmacokinetics and brain uptake of antrafenine

The systemic exposure of antrafenine was studied in non-fasted male C57BL/6 mice weighing 19.8 – 24.6 g. Mice had access to food and water *ad libitum* throughout the pre- and post-dose period. Antrafenine hydrochloride Mw 588.554 (Lot#10-KSS-51-2) was obtained from Toronto Research Chemicals/LGC Standards, Canada, and was dissolved in vehicle; 5% (v/v) DMSO, 25% (v/v) 40% (w/v) Trappsol in 0.9% (w/v) saline, 70% (v/v) 40 mM citrate buffered saline at pH 3.5 at concentrations of 0.5 mg/mL and 1.5 mg/mL.

On the day of dosing, each formulation was prepared by dissolving solid antrafenine in DMSO using vortexing and sonication prior to addition of 40% (w/v) Trappsol in 0.9% (w/v) saline followed by 40 mM citrate-buffered saline at pH 3.5. Each sample was vortexed to create a colorless / light yellow solution and was filtered through a 0.22 µm syringe filter prior to dosing.

Administration of antrafenine was performed by a single bolus IV injection into the tail vein of C57BL/6 male mice using a 1ml syringe with a 25G x 1” needle at a volume of 2 mL/kg. Each

formulation was dosed to mice by bolus injection into the lateral tail vein (2 mL/kg) and blood samples were collected at 1, 2, 5, 15 and 30 min; 1, 2, 4, 7.5 and 24 h post-dose (n=3 mice per time point for each formulation). A maximum of three blood samples were obtained from each mouse, with plasma samples being taken via submandibular bleed (approximately 120 µL). Blood was collected into polypropylene Eppendorf tubes containing heparin as anticoagulant and stabilization cocktail (Complete® (containing a protease inhibitor cocktail and EDTA) and potassium fluoride) to minimize the potential for *ex vivo* compound degradation in blood/plasma samples. Once collected, blood samples were centrifuged immediately, supernatant plasma was removed, snap frozen in dry ice and stored at -80°C until analysis by LC-MS. In addition, at the 15 min, 1, 4 and 24 h post-dose time points, the whole brain was rapidly removed from the carcass soon after the blood collection. The whole brains were blotted to remove excess blood, placed into pre-weighed polypropylene vials and weighed. The brains were snap frozen in dry ice and subsequently stored frozen (-80°C) until analysis.

Bioanalytical method for quantification of Antrafenine by LC-MS

The concentrations of antrafenine in each formulation were determined via a suitably validated generic HPLC-UV assay using a Waters Acquity HPLC system with a Phenomenex Ascentis Express RP-Amide column (50 x 2.1 mm, 2.7 µm) coupled to a Waters PDA detector analyzing at 254 nm. The measured concentrations in each formulation were within 15% of the nominal concentrations, hence the nominal doses were used for data analysis.

Test compound quantitation was performed by comparison of the response to that for a set of calibration standards prepared in the calibration matrix stated in Supplementary Table 5. A stock solution of test compound was spiked into an intermediate solvent containing 50% acetonitrile in water (v/v). Calibration standards were prepared by spiking the calibration matrix with the test compound solution standards and internal standard, maintaining the same final concentration of acetonitrile in all. The extraction from samples and standards was conducted in an equivalent manner using the extraction solvent and ratio described below.

Samples were analyzed on a Waters Xevo TQS Micro coupled to a Waters Acquity UPLC instrument in positive electrospray ionization multiple-reaction monitoring mode for detection, after passing through an ACQUITY UPLC BEH column (50 x 2.1 mm, 1.7 µm) at 40°C with LC conditions of; injection volume of 0.5 µL and a flow rate of 0.8 mL/min. The mobile phase consisted of (A) 0.05% formic acid in water and (B) 0.05% formic acid in acetonitrile delivered by gradient elution from 0 to 95% acetonitrile over 2 minutes. For plasma samples protein precipitation was performed using 80% acetonitrile in water at a 1:5 volume ratio. For brain samples protein precipitation was performed using acetonitrile at a 1:3 volume ratio.

Standards and samples were vortexed, centrifuged and the supernatant was collected for analysis. Analysis was conducted using the instrument conditions described above. Analytical replicates (ARs) were prepared similarly to the calibration standards at three concentrations, and repeat injections of these were included throughout the analytical run to assess assay performance. A summary of the assay validation details is included in Supplementary Table 5.

Supplementary Table 5: Summary of liquid chromatography-mass spectrometry assay validation parameters for quantification of antrafenine in mouse plasma and brain tissue.

Analyte	Antrafenine			
**Matrix**	**Parameter**	**AR (ng/ml)**	**Accuracy (% bias)**	**Precision (%RSD)**
Mouse Plasma	**Accuracy and Precision [a]**	50 (n=6)	1.5	5.4
		500 (n=6)	0.2	6.1
		2000 (n=6)	-1.3	3.6
	**Calibration ^**	**Range (ng/ml)**	**R^2**	**LLQ 9ng/ml)[b]**
		1 - 10000	0.9969	1.0
	**Recovery and matrix factor [c]**	**Recovery (%)**	**Matrix factor (%)**	**Stability**
		108.7	-15.3	Not assessed
^ calibration data were fitted to a linear equation with a weighting factor of 1/x2				
**Matrix**	**Parameter**	**AR (ng/ml)**	**Accuracy (% bias)**	**Precision (%RSD)**
Brain Tissue Homogenate	**Accuracy and Precision [a]**	50 (n=6)	3.2	5.3
		500 (n=6)	0.3	2.2
		2000 (n=6)	-1.2	1.0
	**Calibration ^**	**Range (ng/ml)**	**R^2**	**LLQ 9ng/ml)[b]**
		1 - 5000	0.998	1
	**Recovery and matrix factor [c]**	**Recovery (%)**	**Matrix factor (%)**	**Stability [d]**
		102.2	6.6	107.4
^ calibration data were fitted to a linear equation with a weighting factor of 1/x.				

[a] Acceptance criteria for batch analysis: at least 67% of the AR samples must be within ±15% of nominal values (CDCO In-house acceptance criteria).

[b] The lower limit of quantitation (LLQ) was defined by the lowest acceptable calibration standard for which the back calculated concentration was within ±20% of the nominal concentration.

[c] For plasma, recovery and matrix factor values calculated as an average of four points (10, 50, 500 and 5000 ng/mL). For brain homogenate, recovery and matrix factor and stability values calculated as an average of three technical replicates, at a single point (500 ng/mL).

[d] Stability was assessed for the period of brain sample processing (15 min).

Standard Calculations

The measured concentrations in each of the formulations were within 15% of the nominal concentrations, hence the nominal doses were used for data analysis.

The plasma concentration versus time profile was defined by the average plasma concentration at each sample time, and PK parameters were calculated using non-compartmental methods

(PKSolver Version 2.0). Standard calculations for each pharmacokinetic parameter are listed below.

$$\text{Plasma CL} = \frac{\text{Dose}_{\text{IV}}}{\text{AUC}_{\text{IV,0-inf}}} \qquad \text{Plasma V}_{ss} = \frac{\text{AUMC}_{\text{IV,0-inf}}}{\text{AUC}_{\text{IV,0-inf}}} \times \text{Plasma CL} \qquad t_{1/2} = \frac{Ln(2)}{\lambda_z}$$

CL Clearance in plasma/blood after IV administration

$AUC_{IV,0\text{-}inf}$, Area under the plasma concentration versus time profile from time zero to infinity after IV administration

$t_{1/2}$ Elimination half-life

λ_z Terminal elimination rate constant after IV administration

V_s Apparent volume of distribution in plasma/blood at steady state

$AUMC_{IV,0\text{-}inf}$, Area under the first moment of the plasma concentration versus time profile from time zero to infinity after IV administration

Calculation of Brain Exposure Parameters

The concentration of antrafenine in brain parenchyma was calculated based on the measured concentration in brain homogenate, after correcting for the contribution of compound contained within the vascular space of brain samples as follows:

$$C_{brain} = C_{brain\ homogenate} - C_{brain\ vasculature} \quad \text{where} \quad C_{brain\ vasculature} = C_{plasma} \times V_p$$

C_{brain} = concentration of compound in brain parenchyma (ng/g)

$C_{brain\ homogenate}$ = concentration of compound in brain homogenate (ng/g)

$C_{brain\ vasculature}$ = concentration of compound in brain vasculature (ng/g)

C_{plasma} = concentration of compound in plasma (ng/ml)

V_p = brain plasma volume (17 µL/g for male C57BL/6 mice; Nicolazzo et al, (2010),

Clinical and Experimental Pharmacology and Physiology, **37**, 647-649).

For each mouse, the brain-to-plasma (B:P) concentration ratio (based on total concentrations in each matrix) was calculated as: Brain: plasma = C_{brain} / C_{plasma}. Concentrations in brain were measured as ng/g of tissue, and are presented herein with units of µM, which assumes a sample density of 1 g/mL.

Results

Plasma pharmacokinetics and brain uptake of antrafenine

No adverse reactions or compound-related side effects were observed in any mice following IV administration of antrafenine at 1 and 3 mg/kg. Plasma and brain concentration versus time profiles are presented in Supplementary Fig. 4, with pharmacokinetic parameters and brain

concentrations for individual mice, together with corresponding values for the brain-to-plasma (B:P) ratio are provided in Supplementary Tables 6 and 7, respectively.

Following IV administration at 1 and 3 mg/kg, concentrations of antrafenine in plasma remained measurable for the duration of the 24-hour sampling period, and each profile exhibited an apparent terminal half-life of approximately 7 h. Based on the plasma AUC values, the exposure increased in proportion to the 3-fold increase in dose from 1 to 3 mg/kg. The apparent plasma clearance was low and the volume of distribution was high.

Concentrations of antrafenine in brain were quantifiable at 15 min, 1 and 4 h post-dose, however compound was not detected in brain at 24 h at either dose level. The apparent increase in B:P ratio between 15 min and 4 h suggests that distributional equilibrium between brain and plasma was not achieved instantaneously, and the value at 4 h is likely to provide a better approximation of the steady-state B:P ratio than the values at the earlier two timepoints.

Brain to plasma ratios across the post-dose period were quite consistent at the two dose levels of 1 and 3 mg/kg.

Supplementary Table 6: Pharmacokinetic parameters for antrafenine in male C57BL/6 mice following IV administration.

Parameter	IV administration at 1mg/kg	IV administration at 3mg/kg
**Apparent $t_{1/2}$**	7.3	7.4
**Plasma $AUC_{0\text{-}inf}$ (h*µM)**	1.29	3.14
**Plasma CL (ml/min/kg)**	22.0	27.1
**Plasma VSS (L/kg)**	2.30	3.90

Supplementary Table 7: Individual and mean ± SD (n = 3) plasma and brain concentrations, and brain-to-plasma (B:P) ratios, of antrafenine in male C57BL/6 mice following IV administration at 1 and 3 mg/kg.

Antrafenine (1 mg/kg)							
Time (h)	Mouse ID	Plasma Concentration (µM)		Brain Parenchyma Concentration (µM)		B:P ratio	
		Individual	Mean ± SD	Individual	Mean ± SD	Individual	Mean ± SD
0.25	1	0.313	0.297 ± 0.0569	0.0085	0.0133 ± 0.0075	0.027	0.050 ± 0.039
	2	0.233		0.0220		0.094	
	3	0.344		0.0095		0.028	
1	4	0.122	0.113 ± 0.0089	0.0105	0.0085 ± 0.0029	0.087	0.076 ± 0.027
	5	0.112		0.0052		0.046	
	6	0.104		0.0099		0.096	
4	7	0.0125	0.0131 ± 0.0008	0.0057	0.0039 ± 0.0018	0.46	0.30 ± 0.15
	8	0.014		0.0037		0.26	
	9	0.0129		0.0022		0.17	
24	10	0.0020	0.0018 ± 0.0002	ND	---	---	---
	11	0.0016		ND		---	
	12	0.0018		ND		---	
**Antrafenine (3 mg/kg)**							
Time (h)	Mouse ID	Plasma Concentration (µM)		Brain Parenchyma Concentration (µM)		B:P ratio	
		Individual	Mean ± SD	Individual	Mean ± SD	Individual	Mean ± SD
0.25	13	0.738	0.697 ± 0.0454	0.0311	0.0295 ± 0.0025	0.042	0.042 ± 0.0050
	14	0.705		0.0266		0.038	
	15	0.648		0.0309		0.048	
1	16	0.282	0.280 ± 0.0708	0.0402	0.0314 ± 0.0117	0.14	0.11 ± 0.028
	17	0.208		0.0182		0.087	
	18	0.349		0.0358		0.10	
4	19	0.053	0.0475 ± 0.0059	0.0173	0.0139 ± 0.0030	0.32	0.29 ± 0.039
	20	0.0419		0.0127		0.30	
	21	0.0471		0.0117		0.25	
24	22	0.0057	0.0065 ± 0.0020	ND	---	---	---
	23	0.0088		ND		---	
	24	0.0051		ND		---	

Supplementary Figure 4: Plasma and brain concentrations of antrafenine in male C57BL/6 mice following IV administration at 1 mg/kg (A) and 3 mg/kg (B). The dashed horizontal line represents the minimum detectable concentration of antrafenine.

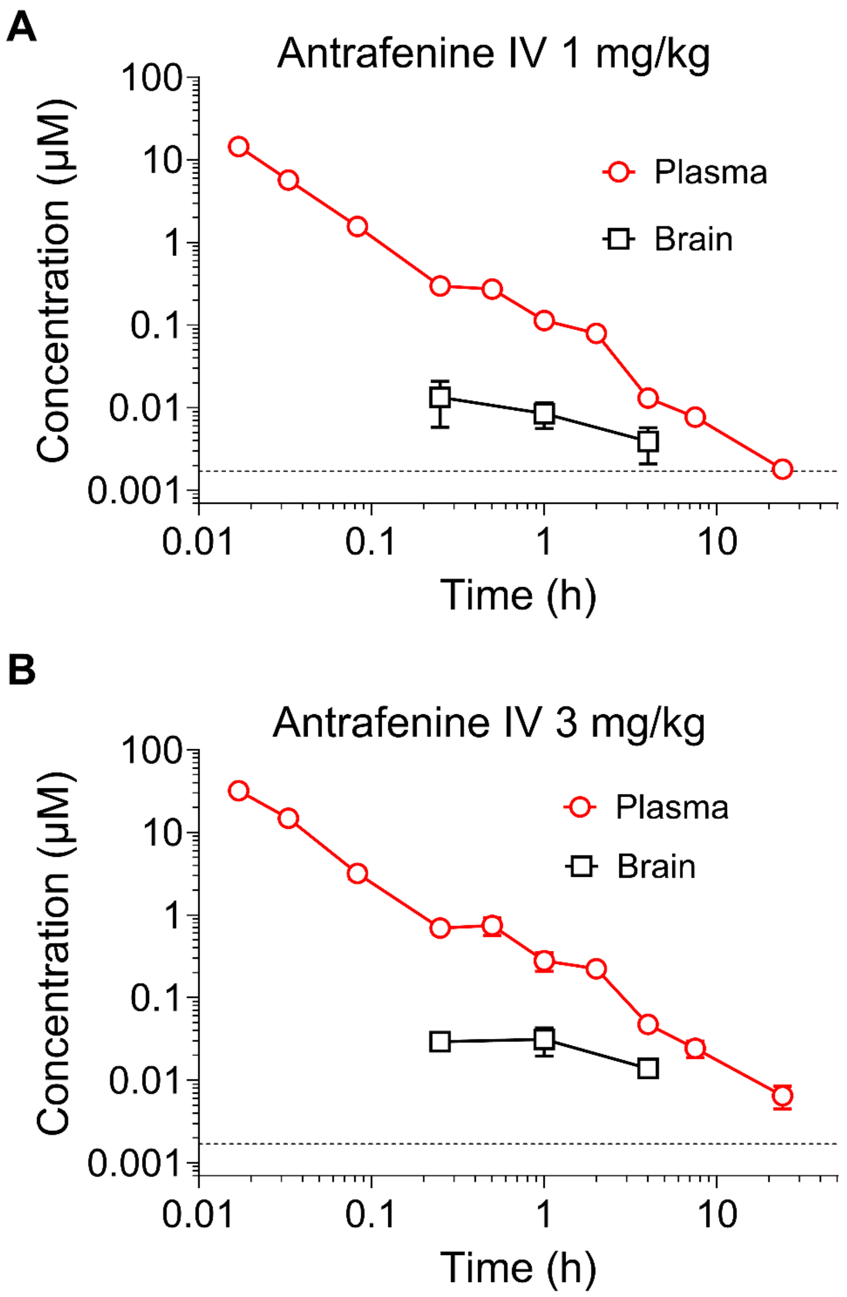

Supplement: Supplementary file 1 — Data S1. Supporting information. [file ANA-98-1261-s001.pdf]
